# The aryl hydrocarbon receptor-interacting protein in cancer and immunity: Beyond a chaperone protein for the dioxin receptor

**DOI:** 10.1016/j.jbc.2024.107157

**Published:** 2024-03-11

**Authors:** Sarah A. Kazzaz, John Tawil, Edward W. Harhaj

**Affiliations:** 1 Department of Microbiology and Immunology, Penn State College of Medicine, Hershey, Pennsylvania, USA; 2 Medical Scientist Training Program, Penn State College of Medicine, Hershey, Pennsylvania, USA

**Keywords:** aryl hydrocarbon receptor-interacting protein (AIP), aryl hydrocarbon receptor (AhR), chaperones, immunity, pituitary adenoma, cancer

## Abstract

The aryl hydrocarbon receptor (AhR)-interacting protein (AIP) is a ubiquitously expressed, immunophilin-like protein best known for its role as a co-chaperone in the AhR-AIP-Hsp90 cytoplasmic complex. In addition to regulating AhR and the xenobiotic response, AIP has been linked to various aspects of cancer and immunity that will be the focus of this review article. Loss-of-function AIP mutations are associated with pituitary adenomas, suggesting that AIP acts as a tumor suppressor in the pituitary gland. However, the tumor suppressor mechanisms of AIP remain unclear, and AIP can exert oncogenic functions in other tissues. While global deletion of AIP in mice yields embryonically lethal cardiac malformations, heterozygote, and tissue-specific conditional AIP knockout mice have revealed various physiological roles of AIP. Emerging studies have established the regulatory roles of AIP in both innate and adaptive immunity. AIP interacts with and inhibits the nuclear translocation of the transcription factor IRF7 to inhibit type I interferon production. AIP also interacts with the CARMA1-BCL10-MALT1 complex in T cells to enhance IKK/NF-κB signaling and T cell activation. Taken together, AIP has diverse functions that vary considerably depending on the client protein, the tissue, and the species.

Chaperone proteins serve an important role in cellular and biological processes as they function to stabilize, maintain the physiological structure, and regulate the subcellular localization of their client proteins (1, 2). For example, the FK506 binding proteins (FKBP), a subfamily of the immunophilins, regulate steroid receptor signaling, and the heat shock response. They bind to and mediate the action of the immunosuppressant drugs cyclosporine, FK506, and rapamycin (3, 4). FKBPs display peptidyl propyl *cis-trans* isomerase (PPIase) activity and possess tetratricopeptide repeat (TPR) domains, 34 amino acid repeats that form a primarily hydrophobic alpha-helical structure that assists in protein-protein interaction (4, 5).

The aryl hydrocarbon receptor (AhR)-interacting protein (AIP, also known as XAP2, ARA9, and FKBP37) is a highly conserved, ubiquitously expressed, cytoplasmic 330-amino acid protein that shares 52% structural similarity with FKBP12 (3, 6, 7, 8). AIP has an amino-terminal PPIase domain that is linked to three TPRs through a 12-amino acid hinge and ends in a terminal α-helix (4, 9). While AIP is similar to FKBPs, it does not bind FK506 or rapamycin and does not display any PPIase-like activity (10, 11, 12).

AIP has been identified as a chaperone for over 20 different proteins (13) but was initially discovered as a binding partner in a yeast 2-hybrid screen of the hepatitis B virus X protein (14). Subsequently, a yeast 2-hybrid screen that used AhR as bait identified the same protein which was named Ah receptor-associated protein (ARA9) (3). While most studies have focused on how AIP modulates the signal transduction pathway of AhR, others have identified novel roles of AIP based on its various other binding partners.

AIP has been proposed to function as a tumor suppressor gene, and mutations in AIP are correlated with 30% of familial isolated pituitary adenomas (FIPAs) and 3% of cases of sporadic pituitary adenomas (15). Most of the pituitary adenomas have a young age of onset and secrete growth hormone (GH) only or both GH and prolactin. These tumors tend to be more aggressive, with large and invasive pituitary adenomas that display resistance to somatostatin analog treatment (7). In addition to endocrine tumors, AIP mutations have also been associated with several other types of cancer, including colorectal cancer, gastric carcinoma, pancreatic carcinoma, and diffuse large B-cell lymphoma (16, 17, 18, 19).

Further investigation into the pathogenicity of AIP mutations in these cancers suggests that AIP can function as either a tumor suppressor or an oncogene depending on the type of cancer (20). These and other scientific studies have determined that the roles of AIP are most likely cell-, tissue-, and species-specific. In this review, we will discuss the canonical functions of AIP as a chaperone protein for its various binding partners, its functions in murine models, the oncogenic and tumor suppressor functions of AIP in different tumors, and its newly discovered roles in modulating the immune system.

## AIP regulates the stability of the AhR cytosolic complex

**Figure 1:**
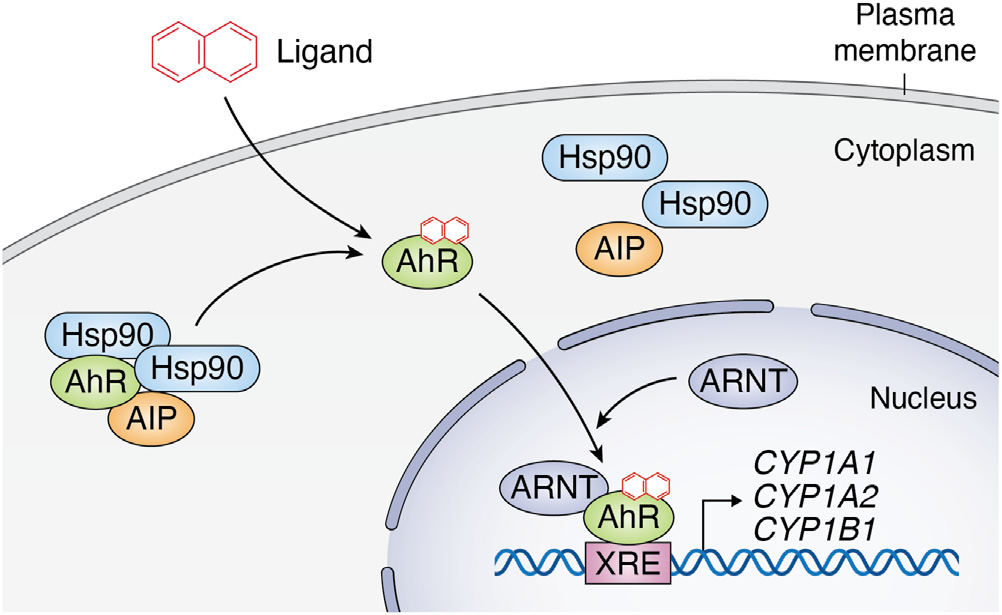
**AhR activation pathway.** Inactivated AhR is found in the cytoplasm, bound to AIP and a dimer of Hsp90. Upon ligand binding, AhR undergoes a conformational change and sheds its chaperone proteins. AhR then translocates to the nucleus where it binds to the AhR nuclear translocator (ARNT), and xenobiotic response elements (XREs) in gene promoters to upregulate the expression of AhR target genes.

The AIP–AhR interaction has been the focus of many studies. AhR is a ligand-activated transcription factor that mediates the metabolism of several environmental toxins, including polyaromatic hydrocarbons and polyhalogenated aromatic hydrocarbons. In its inactive state, AhR resides in the cytoplasm as part of a complex with the heat shock protein 90 (Hsp90) dimer, AIP, and p23 (1). Along with Hsp90, AIP stabilizes AhR in its inactive state and prevents AhR proteasomal degradation. Upon ligand binding (the most potent of which is 2,3,7,8-tetrachlordibenzo*-p*-dioxin, TCDD), AhR sheds its chaperone proteins and moves into the nucleus (8). AhR then interacts with the AhR nuclear translocator (ARNT) and binds to the xenobiotic response element (XRE) in gene promoters to induce the expression of genes regulating the xenobiotic response, including the cytochrome P450 family of metabolic enzymes (Fig. 1) (3).

### The AIP-AhR-Hsp90 complex

**Figure 2:**
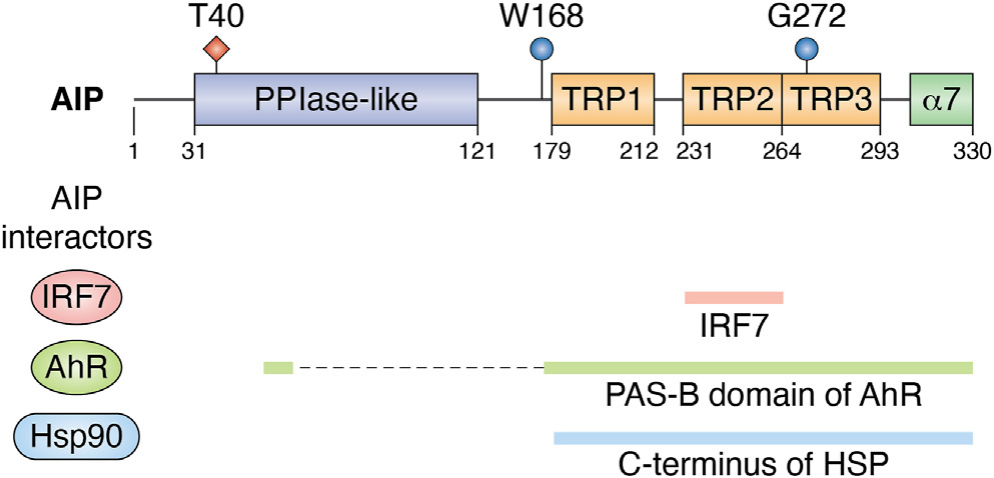
**Schematic of AIP structure.** The peptidyl propyl *cis-trans* isomerase (PPIase) and tetratricopeptide repeat (TPR) domains and the corresponding amino acids are indicated. The areas of AIP that interact with AhR, Hsp90, and IRF7 are indicated in the lines below. The amino acids that are essential for AIP interaction with AhR are highlighted in *blue*. The amino acid (Thr40) that is phosphorylated by TBK1 and increases AIP interaction with IRF7 is indicated in *red*.

AIP is a core component of the AhR cytoplasmic complex, along with Hsp90 (3). Within the complex, AIP interacts with both AhR and Hsp90, suggesting that AIP acts as a brace connecting the two proteins (9). The TPR domains within the C-terminus of AIP are most critical for the Hsp90 and AhR interactions (Fig. 2) (6, 8, 11, 21, 22). In particular, the distal C-terminus (amino acids 311–330) was critical for AIP binding to both AhR and Hsp90 (22). Site-directed mutagenesis studies revealed that AIP Gly272 was necessary for binding to AhR and Hsp90 (21). The recently resolved Cryo-EM structure of the AhR-AIP-Hsp90 complex provided further insight into the interactions (9). Lys66-Lys69 within the PPIase domain, the helix α2-α3 loop within TPR1, and Trp168 of the linker region were identified as the key regions underlying AIP interaction with AhR (9). Although it was thought that the N-terminus of AIP was flexible and did not participate in binding to AhR, cryo-EM analysis revealed that the deletion of the N-terminus of AIP compromised its role in regulating the subcellular location of AhR (22).

AIP has been shown to bind to the carboxyl (C)-terminus of Hsp90 *via* its TPR domain, and mutation of AIP Lys266 to alanine abolished the binding to Hsp90 (8, 11). AIP interaction with AhR is mediated by the Fα helix in the PAS-B domain (C-terminal; amino acids 380–491) and the 40 amino acids that connect the PAS-B domain to the C-terminal transactivation domain (6, 8, 9, 23). Although several residues (*i.e.*, I277, S276, E279, R218) of AhR when mutated to alanine reduced the binding to AIP, other residues including Phe404, Phe406, and Tyr414 were more critical for AIP binding (9).

### AIP as a chaperone for AhR

A number of studies have investigated the biochemical function of AIP within the AhR-Hsp90-AIP cytoplasmic complex. AIP was found to stabilize the unliganded structure of AhR, preventing the ubiquitination and subsequent proteasomal degradation of AhR (1, 7, 8, 9). Consistently, AIP overexpression leads to a two-fold increase in AhR protein levels (5, 23). Therefore, AIP plays a key role as a chaperone in the stabilization of AhR.

AIP was also found to be critical for the cytoplasmic localization and retention of AhR. When overexpressed alone, AhR was found in the nucleus; however, when co-expressed with AIP, AhR was cytoplasmic (24). Furthermore, several studies have shown that AIP delays TCDD-induced nuclear translocation of AhR. AIP was proposed to cause a conformational change in inactive AhR that impedes importin-β binding to the nuclear localization signal of AhR, which can be reversed with ligand binding (5, 7, 8, 23, 25).

Overexpression of AIP was shown to increase the transcriptional response of AhR to dioxin, increasing both the maximal response and the sensitivity of the receptor (6, 8, 26). AIP could also increase the activity of AhR in a dose-dependent manner in a luciferase reporter assay (4).

Other studies examined endogenous levels of AIP and/or AhR in contrast to the previously described overexpression experiments. In Hepa1 cells, AIP expression did not affect endogenous levels of AhR and had minimal to no effect on AhR subcellular localization (25). An AhR point mutant (Tyr408A) was generated that impaired binding to AIP but did not affect binding to ligand or Hsp90. It was found that AIP was essential for the cytoplasmic localization of AhR but repressed the transcriptional activation of AhR (27). Other studies examined species- and cell-specific differences in the regulation of AhR by AIP (28). In comparing mouse and human AhR, it was found that human and mouse AhR proteins exhibited differences in their subcellular localization and nucleocytoplasmic shuttling and regulation by AIP, and AIP remained in a complex with ligand-bound human AhR during shuttling to the nucleus (29).

### Other AIP client proteins

In addition to AhR and Hsp90, AIP has been found to interact with and regulate the function of many of its client proteins (Table 1). AIP binds to multiple heat shock proteins and receptors, including the glucocorticoid receptor (GR), estrogen receptor alpha (ERα), and thyroid receptor β1 (TRβ1), and regulates the functions of these hormone receptors (30, 31, 32). The interactions of AIP with the receptor tyrosine kinase RET (rearranged during transfection), G proteins, and phosphodiesterases were shown to influence cell proliferation and cAMP levels with implications for pituitary tumorigenesis (33, 34, 35, 36, 37). AIP also interacts with and enhances the stability of survivin, a member of the inhibitor of apoptosis (IAP) family, thus providing a potential mechanism for AIP in tumorigenesis (38, 39). AIP may also potentially interact with the cardiac-specific kinase TNNI3K (cardiac troponin I-interacting kinase), which could contribute to the cardiac malformations that occur in AIP knockout mice (13, 40).

**Table 1:** Client proteins of AIP

Client protein	Class of protein	Functional?	Effect	References
Hsp90	Heat shock protein	No	N/A	(1, 11)
Hsc70	Heat shock protein	No	N/A	(94)
HSPA5	Heat shock protein	Not studied	N/A	(84)
HSPA9	Heat shock protein	Not studied	N/A	(84)
HSPA8	Heat shock protein	Not studied	N/A	(94)
HSP90AA1	Heat shock protein	Not studied	N/A	(50)
HSP90AB1	Heat shock protein	Not studied	N/A	(50)
NME1	Cytoskeleton	Not studied	N/A	(84)
TUBB1	Cytoskeleton	Yes	Upregulated with AIP mutants	(84)
TUBB2B	Cytoskeleton	Yes	Upregulated with AIP mutants	(84)
AhR	Cytosolic Receptor	Yes	Expression, localization, and transcriptional activity	(1, 3, 5, 6, 8, 21, 22, 23, 24, 25, 26, 27, 28, 29)
PDE4A5	PDE	Yes	Inhibitory	(36)
PDE2A3	PDE	No	N/A	(35)
ERα	Nuclear Receptor	Yes	Inhibits transcriptional activity	(31)
GR (*via* Hsp90)	Nuclear Receptor	Yes	Cytosolic retention	(7, 12)
PR	Nuclear Receptor	Yes	Reduced transcriptional activity	(7)
PPARα	Nuclear Receptor	Yes	Inhibitory	(83, 95)
TRβ1	Nuclear Receptor	Yes	T3 independent for TR β1	(32)
RET	Transmembrane Receptor	Yes	RET prevents AIP-survivin interaction	(33, 34)
EGFR^a^	Transmembrane receptor	No	N/A	(96)
Gα_q_	G protein	No	N/A	(37)
Gα_13_	G protein	Yes	Inhibits AIP-AHR interaction	(37)
PRKAR1A	Protein kinase	Yes	Inhibitory	(36)
PRKACA	Protein kinase	Yes	Inhibitory	(36)
PDE4A5	PDE	Yes	Inhibitory	(36)
PDE2A3	PDE	No	N/A	(35)
X-protein of HBV	Viral Protein	Yes	Inhibited transcriptional activity	(14)
EBNA-3	Viral Protein	Yes	Nuclear Translocation	(49, 97)
IRF7	Immune	Yes	Cytosolic Retention	(52, 53)
BCL6	Immune	Yes	Prevents degradation	(19)
CARMA1	Immune	Yes	CBM complex formation	(57)
SOD1	Metalloproteins	Not studied	N/A	(84)
AGO1	RISC endonuclease	Not studied	N/A	(50)
LAMP1	Glycoprotein	Not studied	N/A	(98)
LRP1B	Lipoprotein	Not studied	N/A	(99)
TOM20	Mitochondrial	Yes	Promotes preproteins into mitochondria	(39, 94)
Survivin	Mitochondrial	Yes	Stabilizes and promotes mitochondrial transport	(38, 39)
HSPA9	Mitochondrial	Not studied	N/A	(98)
TNNI3K^a^	Cardiac	No	N/A	(40)

Abbreviations: AGO1, Argonaute RISC catalytic component 1; EBNA-3, Epstein-Barr Virus Nuclear Antigen 3; EGFR, epidermal growth factor; ERα, estrogen receptor-alpha; GR, Glucocorticoid receptor; HBV, hepatitis B virus; HSPA8, Heat shock cognate 71 kDa protein; HSPA9, stress-70 protein; HSP90AA1, heat shock protein 90-alpha; HSP90AB1, heat shock protein 90-beta; IRF7, interferon regulatory factor 7; LAMP1, lysosome-associated membrane glycoprotein 1; LRP1B, Low-density lipoprotein receptor-related protein 1B; NME1, NMe/NM23 nucleoside diphosphate kinase 1; PPARα, Peroxisome Proliferator-activated receptor alpha; PR, Progesterone receptor; RET, Rearranged during transfection; SOD1, superoxide dismutase 1; TR β1, Thyroid hormone receptor-β1.

a Indicates further confirmation needed to validate the interaction.

## The physiological roles of AIP learned from murine models

### Cardiovascular development

Mice with a null allele of AIP are not viable with embryonic lethality starting as early as e10 and no embryos surviving past e18.5 (41). These embryos had severe cardiac malformations, including double outlet right ventricle (both the pulmonary artery and the aorta arise from the right ventricle), ventricular septal defect, and pericardial edema. The embryos also exhibited a decreased number and caliber of blood vessels in yolk sacks, petechiae (pinpoint, round spots under the skin that are caused by bleeding), hemorrhaging, and open abdominal cavities (omphalocele) (41). These defects illustrate the critical roles of AIP in cardiac development, which is independent of its role as a chaperone protein for AhR. Mice with a hypomorphic allele of AIP (*Ara9*^fxneo^), which express ∼10% of AIP compared to the expression in wild-type (WT) mice, were also generated (42). *Aip*^*fxneo/fxneo*^ and *Aip*^*+/−*^ mice had significantly decreased liver weights compared to *Aip*^*+/+*^ *and Aip*^*+/fxneo*^ mice. These mice developed patent *ductus venous* (DV), which results in a decreased blood supply to the liver, a phenotype also exhibited by *Ahr* and *Arnt* null mice (42). Therefore, low levels of AIP expression can rescue the lethal impairment of heart development that occurs in *Aip* null mice, but this results in diminished AhR signaling during development, thus phenocopying *AhR*^−/−^ mice.

### Hepatotoxicity

To determine the physiological roles of AIP in the liver, hepatocyte-specific *Aip*^*−/−*^ mice were generated by crossing conditional *Aip*^fx/fx^ mice with mice expressing a *Cre* transgene driven by the albumin (Alb) promoter (43). Despite a 60% reduction in AhR levels, *Aip* null hepatocytes had similar *CYP1A1* and *CYP1A2* expression compared to WT hepatocytes after mice were injected with dioxin. However, *CYP1B1* expression in *Aip* null hepatocytes was reduced in response to dioxin, and mice lacking AIP in hepatocytes displayed resistance to dioxin-induced hepatotoxicity consisting of hepatocellular hydrophobic degeneration and focal inflammation with macrophages, lymphocytes, and necrotic cells (43).

### Pituitary adenoma

Since germline mutations in AIP are associated with the development of pituitary adenomas, two different mouse models were used to investigate the role of AIP in pituitary tumorigenesis (44, 45). Raitila *et al.* (44) found that heterozygous *Aip* (*Aip*^+/−^) mice were significantly more prone to developing pituitary tumors, which were GH-secreting and displayed a more aggressive disease profile. They found that 78% (69/88) of *Aip*^+/−^ mice developed at least one pituitary adenoma by 12 months of age, and some of these mice developed multiple tumors by 6 months of age. Of the 69 pituitary tumors in *Aip*^+/−^ mice, 61 (88%) were GH-secreting. This is compared to 21% (12/58) of the *Aip*^+/+^ littermates by 12 months and most of these tumors (92.6%) were prolactinomas (44). However, in a subsequent study by an independent group, it was found that male *Aip*^+/−^ mice did not develop pituitary adenomas up to the age of 12 months (15). Furthermore, *Aip*^+/−^ mice did not exhibit gigantism/acromegaly (15). At 12 months of age, there was significantly higher total GH secretion in *Aip*^+/−^ mice, but no other abnormalities of GH secretion. Somatotrophs from *Aip*^+/−^ mice had an increased proliferative rate, but this was associated with pituitary hyperplasia rather than any neoplastic changes (15). The reason for the discrepancies between these two studies using the same mouse model is unknown but could be due to environmental factors.

To determine the role of the PKA pathway in AIP-dependent pituitary tumorigenesis, *Aip*^+/−^ mice were crossed with *Prkar1a* (a regulatory subunit of PKA) heterozygote mice (46). Although *Aip*^+/−^ mice displayed signs of acromegaly and elevated insulin-like growth factor 1 levels at 12 months of age, this was not observed in mice heterozygous for both *Aip* and *Prkar1a* (46). However, there were no signs of pituitary hyperplasia or tumorigenesis in either group of mice (46). Thus, AIP regulation of the PKA pathway appears to play an important role in GH secretion.

An independent study generated conditional knockout mice lacking *Aip* in pituitary somatotrophs (sAIPKO mice) (45). Differences in body weight and mean GH hormone levels were greater in the sAIPKO mice compared to WT mice, and the differences were greater between females than males. By 18 weeks of age, sAIPKO mice had enlarged pituitary glands and tumors were visible by 24 weeks, and by 30 weeks of age 80% of sAIPKO mice had tumors (45). Similar to pituitary adenomas in humans that are associated with loss of function *AIP* mutations and display increased tumor invasion and poor response to somatostatin analog treatment, sAIPKO mice had pituitary adenomas that were aggressive and treatment-resistant (45). The cyclin-dependent kinase inhibitor p27 was cytosolic, rather than nuclear, and CDK4 (which mediates the G1-S transition) was perinuclear in siAIPKO pituitary tumors (45). Therefore, loss of somatotroph AIP disrupts the proper regulation of cell cycle regulators which could lead to hyperplasia and pituitary adenomatous transformation.

## Functions of AIP in the immune system

### Binding to viral proteins

Originally, AIP (then termed XAP2) was identified as a binding partner of the X protein of hepatitis B virus (HBV) (14). HBV X acts as a transcriptional activator and is thought to contribute to HBV-induced tumorigenesis (47). AIP binds to the N-terminus (amino acids 13–26) of the X protein, part of the regulatory domain that represses transactivation. The functional outcome of AIP binding to X is repression of the transcriptional activity of the X protein (14).

Epstein–Barr Virus (EBV) nuclear antigen-3 (EBNA-3) is an EBV-encoded nuclear antigen that is necessary for B-cell transformation and one of six nuclear antigens expressed in EBV immortalized lymphoblastoid cell lines (48). A yeast 2-hybrid screen conducted with EBNA-3 as bait using an EBV-transformed human lymphocyte cDNA library yielded AIP (XAP2) as an interacting partner of EBNA-3 (49). Interestingly, the subcellular localization of AIP is modulated by EBNA-3 since AIP is normally cytoplasmic but translocates to the nucleus upon EBNA-3 expression (49).

### Innate immunity

**Figure 3:**
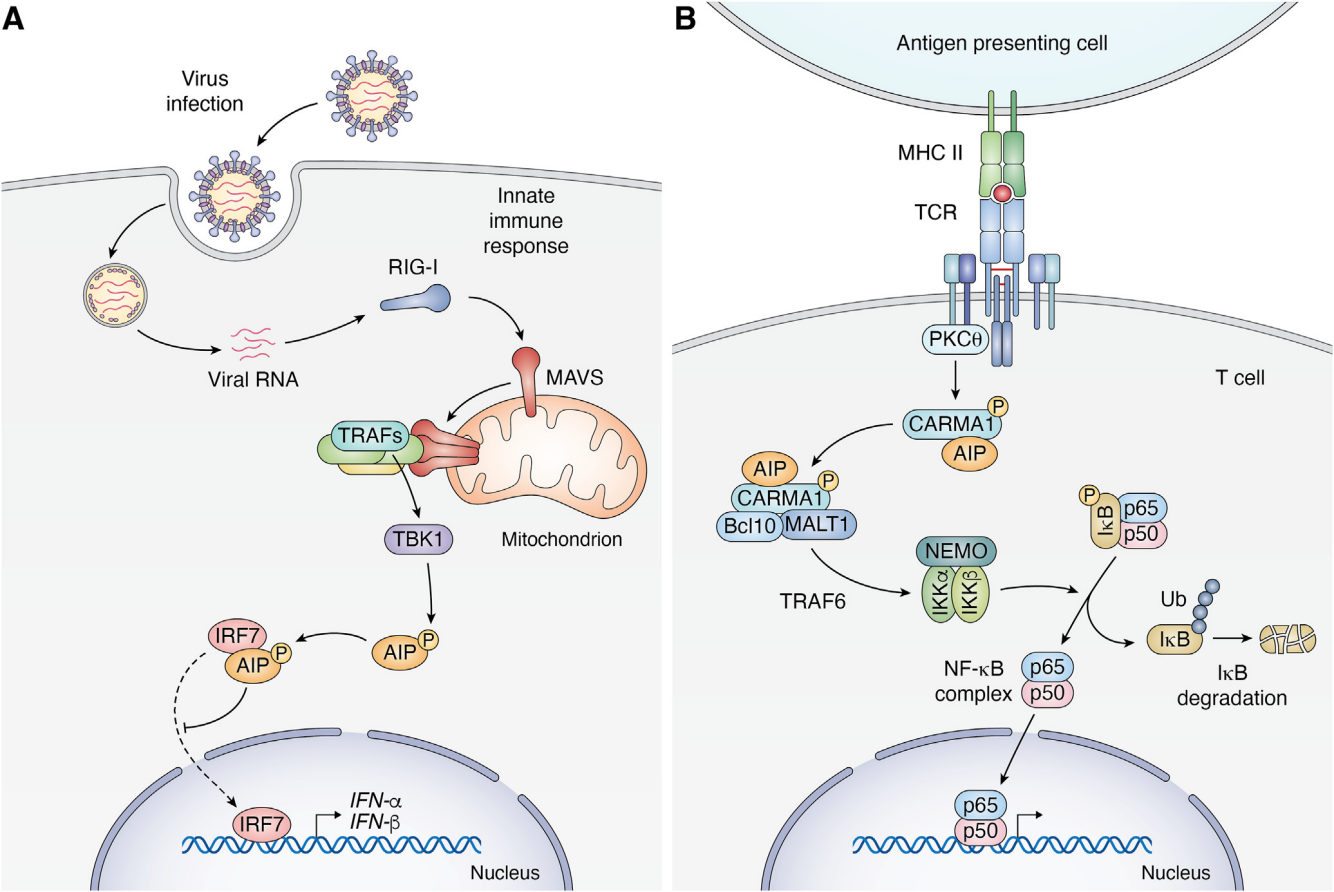
**AIP regulation of innate and adaptive immune signaling pathways.***A*, in the RIG-I-like receptor (RLR) pathway, RIG-I senses 5′-triphosphate double-stranded RNA genomes from RNA viruses, and activates the mitochondrial adaptor MAVS (mitochondrial anti-viral signaling molecule). Activated MAVS forms large prion-like aggregates that recruit TRAF E3 ubiquitin ligases (TRAF2, 3, 5, 6) leading to TBK1 activation. TBK1 phosphorylates AIP at Thr40, which serves as a molecular switch for AIP binding to IRF7 and preventing its nuclear translocation. The AIP-IRF7 interaction suppresses the expression of type I IFNs and interferon-stimulated genes. *B*, following T-cell receptor activation, PKCθ is activated and phosphorylates CARMA1. AIP interacts with and stabilizes CARMA1 in its open conformation, thus enhancing the complex formation of CARMA1 with BCL10 and MALT1. The activated CBM complex triggers IKK and NF-κB signaling.

AIP was identified as a potential binding partner for the transcription factor interferon (IFN) regulatory factor 7 (IRF7) by a proteomics approach mapping the innate immune interactome following virus infection (50). IRF7 is known as the master regulator of type I IFN and is critical for immunity against virus infection (51). The binding of AIP and IRF7 was validated and shown to increase during virus infection (52). The second TPR domain of AIP (amino acids 231–264) mediates the interaction with the virus-activated domain of IRF7 (Fig. 2). AIP functions as an inhibitor of type I IFN production by sequestering IRF7 in the cytoplasm and blocking virus-induced IRF7 nuclear translocation (52, 53) (Fig. 3*A*).

Our recent study demonstrated that AIP is phosphorylated by the kinase TBK1 at Thr40 (Fig. 3) and this acts as a molecular switch to promote the interaction between AIP and IRF7 (53). TBK1 is activated by virus-triggered nucleic acid sensing pathways and therefore plays important roles in both the induction and inhibition of type I IFN activation (54). An AIP T40E phospho-mimetic exhibited enhanced binding to IRF7 and was associated with less type I IFN expression during RNA virus infection (53). Conversely, an AIP phospho-mutant T40A was impaired for IRF7 binding which resulted in increased type I IFN expression and decreased viral replication (53).

### Adaptive immunity

Following T cell receptor co-stimulation with CD28, the signaling scaffold protein CARMA1 (also known as CARD11) is phosphorylated by protein kinase C theta (PKCθ) (55). CARMA1 then forms a complex with BCL-10 and MALT1 (known as the CBM complex), which then activates IκB kinase (IKK) and NF-κB signaling (56). A yeast 2-hybrid screen using the C-terminus of CARMA1 as bait followed by co-immunoprecipitation experiments revealed that the PPI domain of AIP binds to the C-terminus of CARMA1 (57). AIP knockdown in T cells decreases the CARMA1-BCL10 interaction following T cell receptor activation, leading to decreased IKK activity, downstream NF-κB signaling, and IL-2 expression and secretion (57). This indicates that AIP is necessary for proper CBM complex formation, and acts as a positive regulator for NF-κB and NF-AT signaling in activated T cells (Fig. 3*B*).

Within germinal centers, B cells undergo rapid proliferation, class switch recombination, somatic hypermutation, and affinity maturation to generate effective adaptive immune responses. BCL6 is a transcription factor and master regulator of the germinal center B cell phenotype, repressing apoptotic proteins and allowing for continued proliferation of B cells within the dark zone of germinal centers (58). BCL6 can undergo proteasomal degradation triggered by the E3 ubiquitin ligase FBXO11; however, the de-ubiquitinating enzyme UCHL1 binds to BCL6 and protects it from degradation (19, 59). AIP can interact with BCL6, UCLH1, and FBXO11 and displaces FBXO11 from the complex while promoting UCLH1 binding to BCL6, leading to the de-ubiquitination and stabilization of BCL6 (19). Knockout of AIP resulted in decreased BCL6 expression and reduced cell viability of a diffuse large B cell lymphoma (DLBCL) cell line (19). Therefore, AIP acts as a positive regulator of BCL6 and may be involved in the development of DLBCL.

## AIP in tumorigenesis

Loss of function *AIP* mutations have widely been associated with pituitary adenomas, suggesting that AIP is a tumor suppressor in the pituitary gland (7). However, AIP can function as an oncogene in DLBCL and various cancers of the gastrointestinal tract (17, 18, 19, 20). These studies highlight cell-specific roles of AIP in either the prevention or promotion of tumorigenesis.

### AIP mutations are associated with endocrine tumors

Pituitary adenomas are relatively common tumors (prevalence of 1:1000), accounting for about 15% of intracranial tumors (60). While pituitary adenomas are benign, they can be hormone-secreting functional tumors and large, invasive tumors leading to mass effect and compression complications (61). While most pituitary adenomas arise from sporadic mutations, approximately 5% are due to inherited mutations and are considered familial isolated pituitary adenomas (FIPAs). These can be caused by germline mutations in *MEN1*, *GNAS*, or *PRKAR1* leading to multiple endocrine neoplasia type I (MEN1), McCune-Albright syndrome, and Carney complex, respectively (62). However, mutations in *AIP*, located only 2.4 megabases from the *MEN1* gene locus on chr11q13 (63, 64), have been associated with 15% to 30% of FIPA cases and 1% to 3% of sporadic pituitary adenomas (13, 65). Patients with germline AIP mutations have a male predominance and have a younger onset of pituitary adenomas (66). These pituitary adenomas are usually functional, secreting GH (80%) or both GH and prolactin. Pituitary tumors with *AIP* mutations tend to be larger, more invasive, and can present with pituitary apoplexy (13, 67, 68). These tumors are usually resistant to octreotide, a somatostatin analog that is the standard treatment for functional pituitary adenomas (67).

The various *AIP* mutations associated with pituitary adenomas are listed in Table 2. A premature stop codon at Arg304 is the most common mutation, followed by R271W and R16H (69, 70). While there is a small founder effect for R304 in central Italy, R304 is located near a CpG island and is largely considered a hot spot mutation (69). The AIP R304X variant was shown to be less stable than WT AIP and more likely to be ubiquitinated by the F-box-containing protein, FBX03 (71). Most *AIP* mutations often display loss of heterozygosity, low immunostaining, and/or low mRNA levels of AIP (72). In fact, low AIP expression was a stronger predictor of cell proliferation and tumor invasiveness than traditional measures like Ki67 (73). In addition, the half-life of AIP variants directly correlated with the age of diagnosis in pituitary adenoma patients where short AIP variant half-life was observed in patients that were diagnosed at a younger age (71). Taken together, these collective studies suggest that loss of AIP is associated with pituitary tumorigenesis; however, the penetrance of AIP mutations is about 30% (13, 65).

**Table 2:** Germline AIP mutations found in patients with pituitary adenomas

Mutation					Tumor characteristics				References
Gene mutation	Exon	Predicted protein	Protein domain	LOH	Onset	Size	Hormone status	Response to SSA	
Loss of Function Mutations									
Nonsense Mutations									
*c.40 C>T*	1	Q14X	PPIase	Yes	Young	Macro	GH, GH/PRL, PRL, NFPA		(60, 61, 100, 101, 102)
*c.64 C>T*	1	R22X	PPIase		Young	Macro	GH		(100)
*c*.*70G>T*	1	E24X	PPIase		Young	Macro, Micro	GH		(76)
*c.241 C>T*	2	R81X	PPIase		Young	Macro	GH		(76)
*c.424 C>T*		Q142X	PPIase/TPR1		Young	Macro	GH, PRL		(70, 103)
*c.490 C>T*	3	Q164X	PPIase/TPR1		Young	Macro	GH/PRL		(64)
*c.550 C>T*		Q184X	TPR1		Young	Macro	GH		(70)
*c.601A>T*	4	K201X	TPR1		Young	Macro	GH, NFPA		(70, 104)
		E216X	TPR1/TPR2	Yes	Young	Macro	GH		(105)
*c.649C>T*	4	Q217X	TPR1/TPR2		Young	Macro∗	GH/PRL		(103)
*c.662dupC*	4	E222X	TPR2		Young	Macro	GH		(105)
*c.685C>T*	5	Q229X	TPR2		Young	Macro	GH (with papillary thyroid cancer)	Resistant	(106)
*c.715C>T*	5	Q239X	TPR2		Young	Macro	GH		(103)
*c.721A>T*	5	K241X	TPR2		Young	Macro	PRL		(61, 70)
*c.783C>G*	5	Y261X	TPR2		Young	Macro	GH		(104)
*c.804A>C*	6	Y268X	TPR3		Young	Macro	GH, PRL		(61, 107)
*c.910C>T*	6	R304X	α7	Yes	Young	Macro	GH, GH/PRL, PRL		(69, 72, 86, 101, 103, 104, 108, 109)
*c.945C>T*	6	Q315X	α7	Yes	Young	Macro∗	GH, GH/PRL,		(110)
Frameshift Mutation leading to truncated protein									
*c.3_4insC*	1	R2fsX3	PPIase		Young	Macro, Micro	GH, NFPA		(70)
*c.74_81del ins7*	1	L25PfsX130	PPIase		Young	Macro, Micro	GH, GH/PRL, PRL		(64)
*c.88_89delGA*	1	D30T.fsX14	PPIase		Young	Macro	NFPA		(70)
*c.249G>T*	2	Q82fsX7	PPIase	Yes	Young	Macro	GH, PRL		(70, 72)
*c.244_248del* *GAAGG*	2	Q83AfsX15	PPIase		Young	Macro	GH/PRL		(64)
c.286_287 delGT	3	V96PfsX31	PPIase		Young	Macro	GH		(69)
*c.338inc* *ACCC*	3	P114fsX	PPIase		Young	Macro	GH		(70)
*c.350delG*	3	G117A.fsX39	PPIase		Young	Macro	GH, PRL		(61, 70, 86, 104)
*c.404delA*	3	H135L.fsX19	PPIase		Young	Macro	GH		(64)
*c.500delC*		P167HfsX3	TPR1		UNK	UNK	UNK		(70)
*c.517_521del* *GAAGA*	4	Q174fs21X	TPR1	Yes	Young	Macro∗	GH, GH/PRL	Resistant	(72, 103, 111)
*c.543delT*	4	L181fsX13	TPR1		Young	Macro, Micro	GH, PRL, NFPA		(70)
*c.542delT*	4	I182S.fsX12	TPR1						(60, 112)
*c.752delT*	5	L251R.fsX52	TPR2	No	Young	Macro	ACTH		(104)
*c.824_825insA*	6	H275Q.fsX12	TPR3		Young	N/A	GH		(60, 100, 112)
*c.854_857del* *AGGC*	6	Q285.fsX16	TPR3		Young	Macro∗	GH, GH/PRL	Partial	(72, 103)
*c.919insC*	6	Q307fsX104	TPR3/α7		Young	Macro	GH, PRL		(70)
Missense Mutation									
*c.26G>A*	1	R9Q	N-term		Young	Macro, Micro	GH, PRL, ACTH		(104, 113)
*c.47G>A*	1	R16H	N-term	No	Elderly	Macro∗	GH, ACTH	Resistant	(103, 113, 114, 115)
		V49M	PPIase	No	Young		GH		(113, 116)
		W73M	PPIase		Young		GH		(117)
		K103R	PPIase		Young	Micro	ACTH		(91, 113)
*c.512C>T*	4	T171I	TPR1		Young	Macro∗	GH, GH/PRL		(78)
*c.713G>A*	5	C238Y	TPR2	Yes	Young	Macro	GH		(76)
*c.769A>G*		I257	TPR2		Young	Macro	TH		(64, 70)
		G272D	TPR3		Elderly		GH		(84)
*c.896C>T*	6	A299V	TPR3/α7		Young	Micro	GH, PRL		(60, 64, 118)
Promoter and Splice Site Mutations									
*-270_-269 CG > AA; -220 G>A*	5′UTR	Reduced expression		Yes	Young	Macro	GH		(64)
*c.2T>C*	1	No protein			Young	Macro	GH		(70, 86)
*c.807C>T*	6	F269, loss of exon6			Young		GH		(64, 76)
Deletions and Insertions									
*100-1025_279+ 375del*	(ldel) 2	delA34_K39	PPIase		Young	Macro, Micro	GH/PRL/ACTH, GH/PRL		(70)
*1104_-109_279 +578*	(del) 1, 2	Exon1 and 2 deletion	PPIase		Young	Macro	GH		(70)
*Full gene*		Full protein loss			Young	Macro	GH		(70)
*c.794_823dup*	6	Ins274	TPR3		Young		GH		(76)
Function is conserved									
Missense Mutations									
*c.563G>A*	4	R188Q	TPR1		Young	Micro	PRL		(104)
*c.721A>G*	5	K241E	TPR2	Yes	Young	Macro∗	GH, PRL, NFPA	Responsive	(72, 103)
*c.811C>T*	5	R271W	TPR3		Young	Macro, Micro	GH, GH/PRL, PRL		(61, 103)
		E293G	TPR3						(100)
		E293V	TPR3		Elderly		GH		(119)
*c.911G>A*	6	R304Q	α7		Young		ACTH		(60, 76)
Deletions (in frame)									
*c.66-71del* *AGGAGA*	1	delG23_E24	N-term	Yes	Young	Macro	GH		(60, 70)
*c.138_161del24*	1	delG47_R54	PPIase		Young	Macro	GH		(70)
*c.878_879* *AG>GT*	6	E293G	TPR3	Yes	Young		GH		(60)
880-891delCTG GCCCAGCC	6	L294_A297del	TPR3	Yes	Young		GH		(60)
Intron and Splice Site Mutations									
IVS1-1G>C; c100-18C>T	Intron2	UNK			Young	Macro∗	PRL	Partial	(61, 100)
c.469-1 G>A	Intron3	UNK			Young	Macro	PRL		(102, 120)
c.469-2 A>G	Intron3	UNK			Young	Macro	GH/PRL		(70, 100, 104, 120)
Silent Mutations									
c.516C>T	4	D172D	PPIase/TPR1				GH, PRL, NFPA		(114)
c.591G>A	5	E197E	TPR1		Young, Elderly	Macro	GH		(70)
c.807C>T	6	F269F	TPR3		Young	Macro	GH, PRL, NFPA		(121)
Unknown Effect on Function									
Missense Mutations									
		I13N	N-term	Yes	Young		GH		(113, 122)
*c.166C>A*	1	R56C	PPIase		Young	Macro	PRL		(61, 70)
*c.174G>C*	2	K58N	PPIase		Young	Macro∗	PRL	Partial	(61, 104)
		L70M	PPIase		Young, Elderly	Macro	GH/PRL, PRL		(61, 70)
*c.250G>A*	2	E84K	PPIase		Young	Macro	GH/PRL		(70)
		R128H	PPIase/TPR1	No	Young	Macro	GH		(72)
		Q164R	PPIase/TPR1		Young		GH		(72)
*c.509T>C*	4	M170T	PPIase/TPR1		Young	Macro	GH		(104)
		V195A	TPR1	Yes	Young	Macro∗	PRL	Resistance	(61, 72, 123)
		Q228K	TPR2	Yes	Young	Macro	GH		(105, 124)
*c.718T>C*	5	C240R	TPR2		Young	Macro	GH		(70)
*c.803A>G*	6	Y268C	TPR3		Young	Macro	PRL		(61, 70)
		A277P	TPR3	Yes	Young	Macro	GH/PRL		(72)
*c.871G>A*	6	V291M	TPR3		Young	Macro	GH/PRL		(70)
*c.872T>A*	6	V291E	TPR3		Young	Macro	GH/PRL		(104)
		R304N	α7		Young	Macro	GH. ACTH		(100)
		Q307R	α7				ACTH		(124)
		R314W	α7		Young		GH		(84)
*c.974G>A*		R325Q	α7	Yes	Young	Macro	PRL		(104)
Deletion, Duplication, Frameshift, and Insertion Mutations									
*c.138_161del24*	2	G47_R54del	PPIase		Young	Macro∗	GH		(103)
*c.286-287delGT*	3	P46fs	PPIase				GH, GH/PRL		(116)
*c.542delT*	4	UNK			Young		GH		(60)
*c.742_744del TAC*	5	Y248del	TPR2	Yes	Young	Macro	GH		(121)
		L294_A297del	TPR3						(100)
*c.805_825dup*	6	F269_H275dup	TPR3		Young	Macro	GH/PRL		(86, 104)
Mutations in non-coding regions									
*c.220G>A*	Intron1	UNK			Young		GH		(76)
*c.279_269 CG>AA*	Intron1	UNK			Young		GH		(76)
*IVS2 279+ 23C>T*	Intron2	UNK			Young	Macro	GH/PRL		(72)
*IVS2-1G>C*	Intron2	UNK			Young		GH		(60)
*IVS3 468+ 16G>T*	Intron3	UNK		Yes	Young	Macro	GH		(72)
*IVS3 468 +15C>T*	Intron3	UNK		Yes	Young	Macro	GH/PRL/ACTH		(72)
*IVS3-1 G>A*	Intron3	UNK					GH		(100)
*IVS3-2 A>G*	Intron3	UNK							(100)
	3′UTR	UNK					NFPA		(114)

Young is defined as <50 years old at age of diagnosis; Macro is defined as macroadenoma (>10 mm); Macro∗ indicates a macroadenoma with invasive features; Micro is defined as microadenoma (<10 mm).

Abbreviations: α7, terminal alpha-7 helix; ACTH, adrenocorticotropic hormone leading to Cushing’s disease; GH, growth hormone; NFPA, non-functioning pituitary adenoma; PPIase, peptidyl propyl *cis-trans* isomerase domain; PRL, prolactin; TH, thyroid hormone; TPR, tetratricopeptide repeat.

Investigations into *AIP* mutations in other endocrine tumors revealed that AIP is not associated with MEN1 and has little to no role in the genesis of non-medullary thyroid cancer (74, 75). Screening of 132 parathyroid carcinomas revealed the *AIP* R304Q missense mutation in two unrelated women (diagnosed >50 years old) (63). Therefore, while *AIP* mutations are rare, they may predispose to parathyroid carcinoma.

### The role of AIP in the development of pituitary tumors

Initial investigations into defining AIP as a tumor suppressor in the pituitary gland focused on cell proliferation and GH secretion in AIP knockout, knockdown, and expression of mutant AIP variants in different cell types including the GH3 pituitary tumor cell line, 293 cells, and human fibroblasts (7). Expression of WT AIP reduced cell proliferation whereas AIP-deficient GH3 cells exhibited increased cell proliferation and AIP mutants were unable to decrease cell proliferation (76, 77, 78). AIP KO GH3 xenografted BALB/c nude mice had tumors that were four times larger than control cells with more GH in secretory vesicles and increased cell proliferation as measured by Ki67 (77). The mice with AIP KO GH3 xenografts also weighed more and were larger (in length and width) 8 weeks after inoculation and had higher GH and IGF-1 levels (77). AIP can be found in association with secretory vesicles in somatotroph (GH-secreting) and lactotroph (PRL-secreting) cells (15). Consistently, AIP-deficient GH3 cells had a 7.3-fold increase in GH synthesis and 1.3-fold increase in PRL synthesis (77, 78). This increase in hormone secretion may be mediated by a “leaky” ryanodine receptor which has been shown to cause excessive Ca^2+^ release from the ER in *C. elegans* with non-functional AIP variants (79).

AIP knockdown and AIP variants were also associated with a more invasive phenotype (78). AIP-deficient GH3 cells exhibited epithelial-to-mesenchymal-transition (EMT)-like changes with elongated, spindle-shape mesenchymal morphology, increased flexibility and elevated release of chemotactic factors (80). *AIP*-mutation positive pituitary tumors were associated with an altered tumor microenvironment including increased CD86+ macrophage and FOXP3+ Tregs infiltration, and upregulation of tumor-derived cytokine CCL5 (also known as RANTES). These *AIP*-mutation positive tumors had a gene signature indicative of increased EMT, with downregulation of CDH1 (E-cadherin), absent membranous CTNNB1 (B-cadherin), increased expression of ZEB1 (Zinc-finger E-box binding homeobox 1), and decreased expression of ESPR1 (epithelial splicing regulatory protein 1), PERP (TP53 apoptosis effector), and EPCAM (epithelial cell adhesion molecule). Furthermore, pituitary-specific AIP KO mice had a greater disruption of the reticulin network and increased F4/80+ infiltrating macrophages in the pituitary gland of 15-month-old mice (80). Together, these findings highlight the disruption of the tumor microenvironment in *AIP*-mutant-positive tumors and suggest that CCL5 and its receptor CCR5 could serve as potential therapeutic targets.

Another AIP variant, a T171I missense mutation, was found in three patients with pituitary adenoma and was associated with decreased expression of Sstr2 (somatostatin receptor 2), which is needed for responsiveness to somatostatin analogs (78). The AIP variant was also associated with decreased expression of the tumor suppressor gene *Zac1*. In contrast, there was increased phosphorylation of Stat3 and IL-6 expression, a well-characterized oncogenic signaling axis (78).

AIP mutations are generally loss-of-function and have been linked to diminished or impaired protein interactions since about 75% of known mutations are clustered in the C-terminus of AIP, a region essential for most of the AIP protein-protein interactions (13, 45, 81). Indeed, several AIP binding partners, including PPARα and NME1 (a tumor suppressor that negatively regulates cell migration, motility, and proliferation and has a negative correlation with pituitary tumor extension into the cavernous sinus) have been associated with pituitary tumorigenesis (82, 83, 84). However, it was determined that there was no correlation between PPARα and AIP in pituitary tumors (83). Furthermore, the effect of AIP knockdown on GH3 secretion in the presence of AhR ligands was inconclusive (85, 86). The functional effects of the AIP-NME1 interaction remain unknown (84). More work is needed to understand how AIP mutations impact its interactions with other proteins and the effects of those interactions on pituitary tumorigenesis.

**Figure 4:**
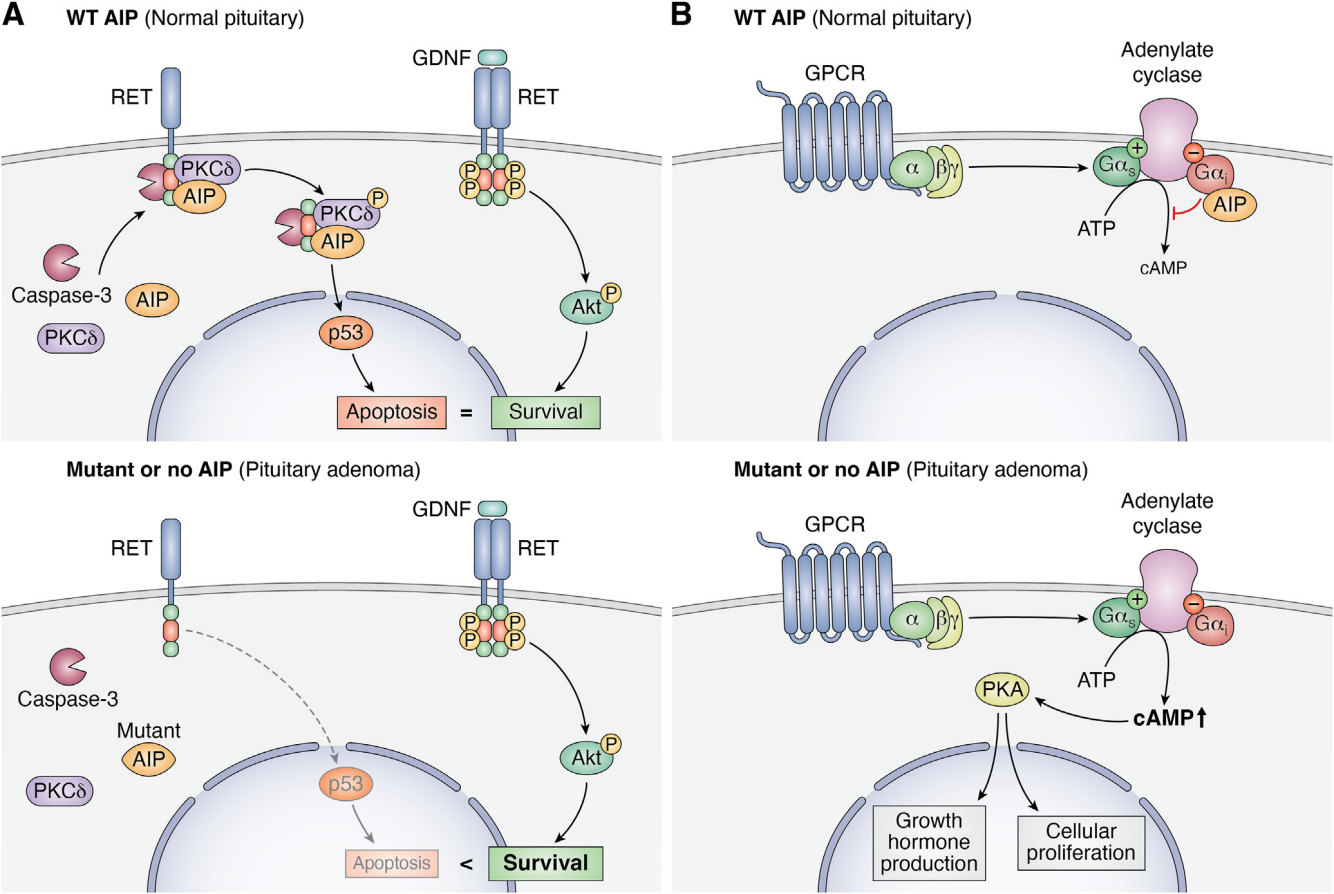
**Tumor suppressor function of AIP in somatotrophs.***A*, AIP regulates RET signaling to promote apoptosis. GDNF (glial cell-derived neurotrophic factor) binding to the RET receptor promotes cell survival through AKT signaling. In wild-type AIP somatotrophs (*top*), AKT cell survival signaling is balanced by the pro-apoptotic RET/caspase-3/PKCδ pathway. In this pathway, AIP promotes the shuttling of caspase-3 and PKCδ to the intracellular (IC) portion of the RET receptor. Caspase-3 cleaves the IC portion of RET and PKCδ which activates the pro-apoptotic p53 pathway. However, in somatotrophs expressing loss of function AIP or no AIP (*bottom*), the RET/caspase-3/PKCδ pathway is downregulated and the pro-survival GDNF-mediated RET/AKT pathway predominates over the pro-apoptotic RET/caspase-3/PKCδ pathway thus predisposing to pituitary adenoma formation. *B*, AIP regulates cAMP signaling through its interaction with the inhibitory Gα_i_ subunit (*top*). When AIP is mutated or deleted, cAMP signaling is dysregulated. The increased cAMP promotes PKA-mediated GH production and cellular proliferation.

In the RET/caspase-3/PKCδ pathway, RET is cleaved and intracellular RET forms a complex with caspase-3 and PKCδ (87). PKCδ is then phosphorylated and cleaved by caspase-3, leading to the phosphorylation of JNK and CREB, which upregulate PIT1, ARF, and p53 to promote apoptosis. AIP stabilizes the RET-caspase 3-PKCδ complex in PIT1-expressing cells (somatotrophs and lactotrophs) promoting the apoptotic PIT1-CDK2N1-ARF-p53 signaling pathway (20, 34), thereby balancing the pro-survival GNDF-mediated activation of RET and the AKT pathway (Fig. 4*A*, *top*). In the absence of AIP or in the presence of AIP pathogenic mutants, the RET/caspase-3/PKCδ complex is disrupted, which leads to increased proliferation of somatotrophs (34) (Fig. 4*A*, *bottom*). However, six common AIP mutants found in patients with pituitary adenoma were still able to interact with RET (33).

Since neuroendocrine tumors often have dysregulated cAMP signaling (68, 88), AIP regulation of cAMP *via* its interactions with PDEs and G-proteins has been the subject of several studies. AIP has been shown to reduce cAMP levels and activity, which was impaired by AIP R304X expression (77, 78, 88). In addition, overexpression of AIP caused a reduction in GH secretion, while AIP knockdown or AIP R304X expression increased GH secretion (88). Furthermore, microarray analysis revealed that *Aip*^*−/−*^ MEFs have impaired cAMP signaling (68). Knockout or knockdown of *AIP* resulted in the accumulation of cAMP, and this was due to defective Gα_i2_ signaling and subsequent downregulation of pERK1/2 and p-CREB (68). Several N-terminal AIP mutants were shown to have disrupted Gα_i-2_ and Gα_i-3_ protein function, subsequently leading to increased cAMP and GH levels, as well as increased cell proliferation (68) (Fig. 4*B*). AIP also interacts with PKA regulatory and catalytic subunits to diminish PKA activity (36). Finally, various AIP mutants were shown to lose the interaction with phosphodiesterase-4A5 (PDE4A5) (76), although it is unclear if this is related to its tumor suppressor functions.

In addition to AIP mutants, downregulated expression of AIP could also promote the development of pituitary adenomas. Known regulators of AIP expression include general transcription factor IIb (GTF2B) and microRNAs (89, 90, 91, 92). There was a positive correlation between GTF2B and AIP mRNA expression, with high-grade GH-secreting pituitary adenomas having both lower GTF2B and AIP expression (89). Two micro-RNAs, miR-107 and miR-34a, have been associated with cancers and decreased AIP expression. While miR-107 binds to the 3′UTR of AIP and downregulates AIP expression, no correlation was observed between AIP levels and miR-107 expression in pituitary adenomas (90). High expression of another microRNA, miR-34a, was also associated with more invasive pituitary adenomas and was shown to decrease AIP expression through regulation of the 3′UTR of AIP (91). More work is needed to understand how AIP expression is normally regulated and how its expression could be impacted during pituitary tumor development.

## AIP in non-endocrine tumors

Elevated AIP expression has been associated with a worse prognosis of several carcinomas of the gastrointestinal tract, including cholangiocarcinoma, pancreatic carcinoma, gastric carcinoma, and colorectal cancer (17, 18, 20). While these studies mainly found a correlation between AIP expression and carcinogenesis, rather than AIP activity, they nevertheless suggest that AIP may act as an oncogene in the gastrointestinal tract.

AIP expression was examined in pancreatic tumors from 204 patients (17). While cytoplasmic AIP expression was associated with a worse prognosis, 9.8% of samples with nuclear AIP staining were associated with a better prognosis (17). However, it remains unclear why nuclear AIP would improve the prognosis of pancreatic cancer patients.

Another study examined AIP expression in 147 cases of gastric cancer, and 36.6% of the tumors had high AIP expression (18). AIP staining was cytoplasmic in most of the tumors. Interestingly, high AIP expression was significantly and independently correlated with tumor progression and death, with high AIP expression associated with lower survival and higher progression rates. This suggests that AIP acts as an oncogene in gastric cancer, but again the mechanism is unknown (18).

Examination of the Cancer Genome Atlas database found that high AIP expression was associated with decreased survival and increased risk of relapse in colorectal cancer. Furthermore, functional studies revealed that AIP overexpression in colon epithelial cells increased cell migration and EMT transition, and inoculation of these cells in a mouse model of metastatic colon cancer led to liver metastasis and decreased survival (20).

AIP overexpression was noted in several highly metastatic colon cancer cell lines (KM12SM, SW620, Lim1213) compared to poorly metastatic cell lines (16). There was also a significant association between high AIP expression and lower survival in a public cohort of 508 colorectal cancer patients. When poorly metastatic colorectal cancer cells were stably transfected with AIP, there was increased activation of SRC, JNK, and AKT kinases and characteristics of the more metastatic, invasive colorectal cancer cell line, KM12SM, including increased migration, EMT, adhesion, and invasion (16). Proteomic analysis in the study further revealed that AIP upregulated the expression of the cell adhesion protein cadherin-17 (CDH17), which may promote liver adhesion and homing of colorectal cancer cells expressing AIP. Inoculation of AIP overexpressing KM12 cells into the spleen of nude mice yielded larger and highly proliferative tumors with increased liver metastasis, resulting in decreased survival (16).

In addition to these GI cancers, high AIP expression is also associated with DLBCL as revealed by the cBioPortal database for Cancer Genomics, histological examination of primary DLBCL compared to healthy controls, and in various DLBCL cell lines (19). As mentioned earlier, AIP involvement in DLBCL may be through its positive regulation of the transcription factor BCL6.

**Figure 5:**
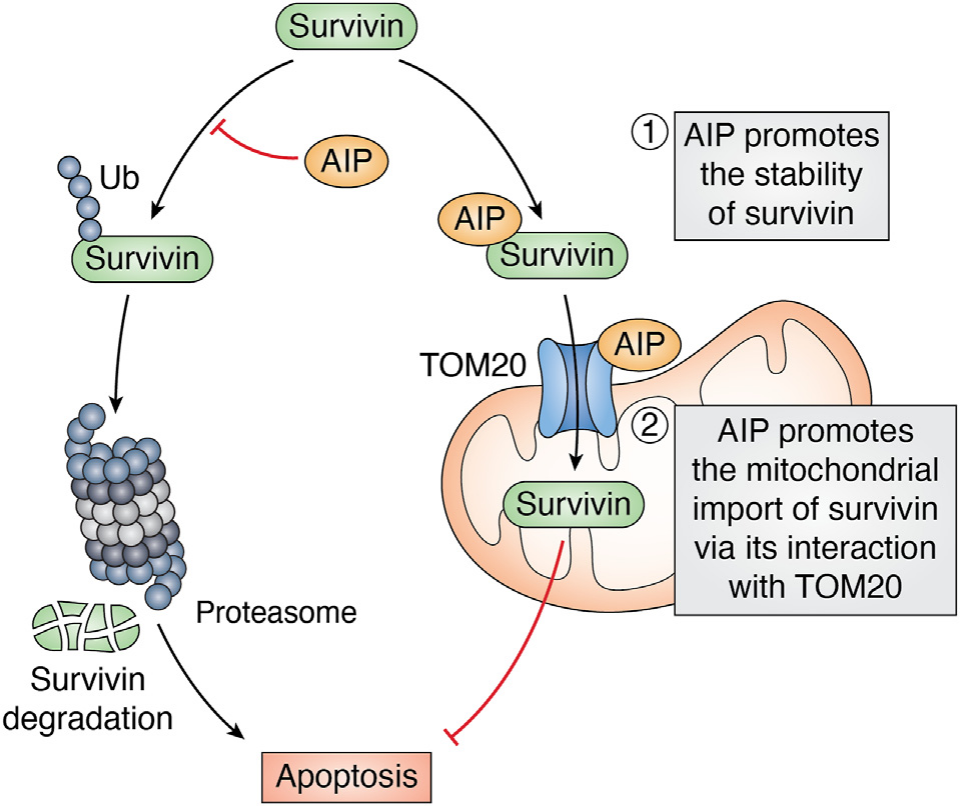
**AIP promotes survivin stability and its mitochondrial import *via* TOM20.** AIP functions as a chaperone to enhance the stability of the anti-apoptotic protein survivin. AIP also interacts with the mitochondrial import receptor TOM20 to facilitate the shuttling of survivin to the mitochondria, where it is imported and exerts its anti-apoptotic functions.

Although the mechanisms of AIP oncogenic functions are not well known and could vary depending on the specific tumor, AIP has been shown to interact with and regulate potential oncogenes. As discussed earlier, a proteomics screen identified AIP as an interacting protein of survivin, a member of the anti-apoptotic IAP family (38). AIP binds to and stabilizes survivin and therefore knockdown of AIP destabilizes survivin and enhances cell death (38). AIP is therefore likely a chaperone protein important to stabilize survivin. Furthermore, through its interaction with the mitochondrial protein, TOM20, AIP promotes the mitochondrial import of survivin to promote cell survival (39) (Fig. 5). AIP regulation of survivin is a salient example of a potential oncogenic mechanism but additional studies are needed to further understand how AIP can promote tumorigenesis.

## Closing remarks

AIP has been widely studied both as a co-chaperone molecule for AhR and as a tumor suppressor in the pituitary gland. However, AIP interacts with many other proteins and exerts a variety of effects on most of its client proteins that can be cell- and species-specific. Further investigations into known AIP-binding proteins and the discovery of novel interacting partners will undoubtedly unveil new functions and physiological roles of AIP, particularly in the immune system.

AIP suppresses the innate immune response by inhibiting IRF7 but enhances T-cell activation through the CBM complex and NF-κB activation. AIP likely has other functions in the immune system that are unexplored, but the generation of AIP conditional knockout mice in different immune cell types could shed light on its novel functions in the immune system. The tissue-specific roles of AIP are further highlighted in its disparate roles as a tumor suppressor in the pituitary gland and an oncogene in the context of DLBCL, colorectal, gastric, and pancreatic cancers (17, 18, 19, 20). AIP tumor suppressor function is likely mediated by its regulation of RET and/or Gα_i_ signaling in somatotrophs, such that loss of function mutations in AIP lead to increased hormone secretion and cell proliferation (33, 34, 35, 36, 37). However, how AIP exerts oncogenic activity remains an open question, although the survivin-AIP interaction could potentially explain its oncogenic functions in certain cancers. Regardless, additional studies are needed for a more refined understanding of how AIP can promote oncogenesis in a tissue-specific manner.

The immune regulatory functions of AIP could potentially contribute to its described roles in cancer. Pituitary-specific deletion of AIP in mice led to increased infiltration of tumors with macrophages and FOXP3+Tregs, consistent with AIP-mutation-positive tumors in humans *versus* sporadic pituitary tumors (80). Increased tumor-associated macrophages were associated with an EMT-like phenotype, caused by macrophage-derived soluble factors (80). Therefore, AIP likely plays an important role in regulating the tumor microenvironment and restricting the recruitment of immunosuppressive tumor-associated macrophages and Tregs. Similar to AIP, IRF7 has been shown to play complex roles in the tumor microenvironment and can exert oncogenic or tumor suppressor activity, depending on the type of cancer (93). It will be of interest to examine IRF7 expression and activation as well as type I IFN expression in the tumor microenvironment of pituitary tumors as well as cancers associated with elevated AIP expression. AIP overexpression in tumors could potentially suppress the innate immune response and type I IFN in the tumor microenvironment which may contribute to the development and/or metastasis of certain cancers. Future studies should investigate how tumor-intrinsic AIP *versus* immune-cell AIP expression impacts the tumor microenvironment and tumor progression.

## Conflict of interest

The authors declare that they have no known competing financial interests or personal relationships that could have appeared to influence the work reported in this paper.
